# Are patients with schizophrenia impaired in processing non-emotional features of human faces?

**DOI:** 10.3389/fpsyg.2013.00529

**Published:** 2013-08-20

**Authors:** Hayley Darke, Joel S. Peterman, Sohee Park, Suresh Sundram, Olivia Carter

**Affiliations:** ^1^School of Psychological Sciences, University of MelbourneParkville, VIC, Australia; ^2^Department of Psychology, Vanderbilt UniversityNashville TN, USA; ^3^Department of Molecular Psychopharmacology, Florey Institute of Neuroscience and Mental HealthParkville, VIC, Australia; ^4^Northern Psychiatry Research CentreEpping, VIC, Australia

**Keywords:** schizophrenia, vision, face, identity, detection, recognition, gait, perception

## Abstract

It is known that individuals with schizophrenia exhibit signs of impaired face processing, however, the exact perceptual and cognitive mechanisms underlying these deficits are yet to be elucidated. One possible source of confusion in the current literature is the methodological and conceptual inconsistencies that can arise from the varied treatment of different aspects of face processing relating to emotional and non-emotional aspects of face perception. This review aims to disentangle the literature by focusing on the performance of patients with schizophrenia in a range of tasks that required processing of non-emotional features of face stimuli (e.g., identity or gender). We also consider the performance of patients on non-face stimuli that share common elements such as familiarity (e.g., cars) and social relevance (e.g., gait). We conclude by exploring whether observed deficits are best considered as “face-specific” and note that further investigation is required to properly assess the potential contribution of more generalized attentional or perceptual impairments.

## Introduction

Face processing deficits have been repeatedly demonstrated in patients with schizophrenia, however, debate continues regarding the precise nature of these impairments and the mechanisms that underlie them. Some authors posit that face processing deficits are specific to emotion-related content (Edwards et al., 2002; Schneider et al., 2006); others argue that they represent an impairment in processing biological and socially relevant stimuli (Kim et al., 2010); a more general impairment in visual attention (Caharel et al., 2007); or a generalized difficulty in processing complex visual stimuli—of which faces are just one example (Doop and Park, 2009). In contrast to the vast literature using tasks involving identification or recognition of facial expression and the relationship to impaired emotion processing (see Kohler et al., 2010), relatively little has been done to systematically assess face processing outside an emotional context in individuals with schizophrenia (c.f. a review in the current issue comparing face recognition deficits in schizophrenia and autism Watson, 2013). Before discussing the existing patient literature we will briefly consider the current theories of face processing more broadly.

## The separation of identity and expression processing

The dominant theory of face perception as first proposed by Bruce and Young (1986) makes a division between identity and emotion recognition representing two largely independent processes. A neuroanatomical framework for this dual route model has since been provided by Haxby et al. (2000) and distinguishes between two types of information: invariant and changeable. Invariant information refers to properties that are consistent across different views and facial expressions, and is necessary for recognizing the identity of a face. Changeable information includes eye gaze, expression, and movements of the eyes and mouth, and is necessary for the recognition of facial affect. The initial processing of facial features is proposed to be mediated by neurons in the inferior occipital gyri (Haxby and Gobbini, 2011). From here, the analysis of “variant” or changeable visual features is processed largely via a route involving the posterior superior temporal sulcus (pSTS). In contrast, invariant (unchangeable) information is proposed to be processed via a ventral temporal route including the inferior occipital and fusiform gyri. These two routes then have differing degrees of connectivity with either limbic or cortical regions outside these face selective areas of visual extrastriate cortex.

Support for this separation of identity and expression recognition comes from a broad range of sources (for review see Calder and Young, 2005) including behavioral research (Bruce, 1986; Campbell, 1996; Calder et al., 2000), functional imaging studies (George et al., 1993; Sergent et al., 1994; Winston et al., 2004), and in dissociations exhibited by individuals with brain injury (Tranel et al., 1988; Young et al., 1993; Hornak et al., 1996) and prosopagnosia (Baudouin and Humphreys, 2006; Riddoch et al., 2008). Compelling neurophysiological evidence for this dissociation has also come from studies of non-human primates (Tsao et al., 2003; Pinsk et al., 2005; Rajimehr et al., 2009) where multiple “patches” of face-selective cortex have been identified that show selectivity to identity or expression processing, respectively (Tsao et al., 2008a,b).

While this evidence demonstrates that identity and expression recognition involve separate processes, the level at which this bifurcation occurs, and the degree to which these parallel processes interact, are yet to be resolved. Importantly, it is also unclear the extent to which identity recognition and emotional processing are inextricably linked (Fitousi and Wenger, 2013; and for review see Calder, 2011). To properly understand the nature of face processing deficits in schizophrenia it is important to consider deficits in emotionally neutral judgments based on either changeable or invariant information.

## Identity processing deficits

**Figure 1:**
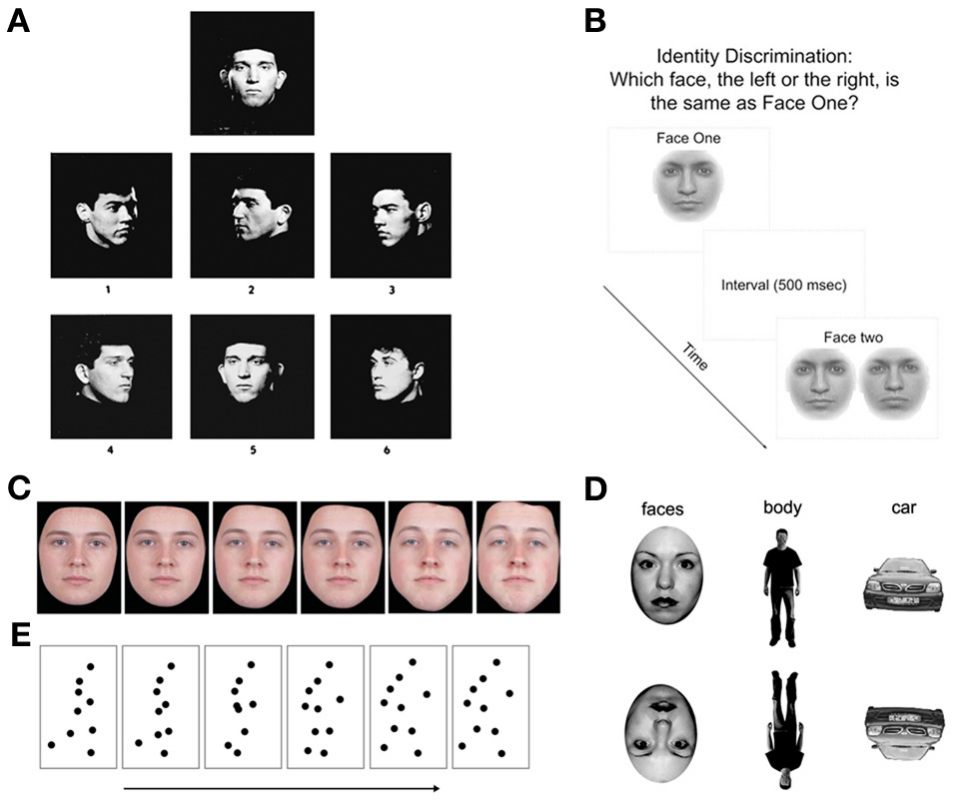
**(A)** Plate from the Benton Facial Recognition Test (Benton, 1983). Participants indicate which of the six images match the target. (Published in Busigny and Rossion, 2010, p. 969). **(B)** Identity matching task used by Norton et al. (2009). **(C)** Example of morphed images ranging from “no sex” (50% male, 50% female) to 100% male face (from Bediou et al., 2005, p. 528). **(D)** Examples of upright and inverted stimuli used in Soria Bauser et al. (2012). **(E)** Example of semi-successive frames of the point-light displays (walking) used in Kim et al. (2005).

Deficits in non-emotional face processing have been demonstrated using a variety of behavioral tasks. However, heterogeneity across tasks types and participants has produced mixed results (see Table 1). Tasks that assess true identity processing are primarily matching tasks, where the participant views static photographs of faces (with non-face identifying features removed, such as hair and spectacles), either serially or concurrently. The participant must match the identity of the first face to one of several options (Addington and Addington, 1998; Penn et al., 2000; Kucharska-Pietura et al., 2005; Chen et al., 2012). The most commonly used face-matching task is the Benton Test of Facial Recognition (Benton, 1983; see Figure 1A). Individuals with schizophrenia have shown impaired performance on the Benton test in many (e.g., Addington and Addington, 1998; Evangeli and Broks, 2000; Whittaker et al., 2001; Hooker and Park, 2002; Kucharska-Pietura et al., 2005; Soria Bauser et al., 2012), but not all studies (Hall et al., 2004; Scholten et al., 2005; Van 't Wout et al., 2007; Pomarol-Clotet et al., 2010). One study using a booklet-based face-matching task with similar properties to the Benton also found no impairment (Hooker and Park, 2002). Three studies using a morphed identity-matching task—in which participants choose which of a pair of faces of varying similarity matches a briefly presented target—have also produced inconsistent results, with two studies reporting no impairment (Norton et al., 2009; Chen et al., 2012) and only one study approaching significance (Chen et al., 2009) (see Figure 1B). The reason for these inconsistent findings is unclear. Given the relatively heterogeneous patient samples, however, the influence of factors such as age, illness duration, and gender may be worth exploring through a formal meta-analysis in the future.

**Table 1:** **Identity recognition tasks used in schizophrenia research from the last 20 years—All results relate to performance of Schizophrenia patients relative to controls**.

**Task**	**Results**	**References**
**TASKS ASSESSING IDENTITY DISCRIMINATION**		
Benton Test of Facial Recognition (see Figure 1A)	Significantly impaired	Borod et al., 1993,^1^; Kerr and Neale, 1993,^1^; Bellack et al., 1996,^2^; Mueser et al., 1996,^1^; Salem et al., 1996,^1^; Addington and Addington, 1998,^1^; Evangeli and Broks, 2000,^1^; Penn et al., 2000,^1^; Whittaker et al., 2001,^2^; Hooker and Park, 2002,^2^; Kucharska-Pietura et al., 2005,^2^; Soria Bauser et al., 2012,^3^ Hall et al., 2004,^1^; Scholten et al., 2005,^3^; Van 't Wout et al., 2007,^1^; Pomarol-Clotet et al., 2010,^3^
	Not significantly impaired	
Morphed-Faces Identity Matching (Match target to 1 of 2 choices)	Approaching significant impairment Not significantly impaired	Chen et al., 2009^*^ Norton et al., 2009; Chen et al., 2012
Neutral Face Recognition Task (Match target to 1 of 7 choices)	Not significantly impaired	Hooker and Park, 2002
Identity Discrimination (same/different judgments of serially presented faces)	Significantly impaired Not significantly impaired	Martin et al., 2005; Butler et al., 2008; Shin et al., 2008^*^; Soria Bauser et al., 2012; Edwards et al., 2001; Johnston et al., 2010; Soria Bauser et al., 2012
Identity Recognition Task (Is this Person A or B?)	Significantly slower (accuracy at ceiling for both groups)	Baudouin et al., 2002
**TASKS ASSESSING ONE ASPECT OF IDENTITY**		
Morphed Sex Recognition Task	Not significantly impaired	Bediou et al., 2005, 2007, 2012
Age Discrimination Task (indicate the age by decade: 1=teens … 7= seventies)	Not significantly impaired—(speed/accuracy trade-off) Significantly impaired	Schneider et al., 1998, 1995; Kohler et al., 2000
Age Discrimination Task (Judge whether face is older or younger than 30)	Not significantly impaired Significantly impaired	Gur et al., 2002a,b; Schneider et al., 2006
**TASKS ASSESSING FAMILIARITY/MEMORY FOR FACES**		
Penn Face Memory Test	Significantly impaired	Sachs et al., 2004; Calkins et al., 2005^*^; Silver et al., 2009
The Warrington Recognition Memory Test–Faces subtest	Significantly impaired Not significantly impaired	Whittaker et al., 2001; Soria Bauser et al., 2012; Evangeli and Broks, 2000
Famous Faces	Significantly impaired Not significantly impaired	Pomarol-Clotet et al., 2010; Evangeli and Broks, 2000; Whittaker et al., 2001; Joshua and Rossell, 2009^*^
Familiarity test for known and unknown people (is this face familiar or unknown?)	Significantly impaired	Caharel et al., 2007

1 *Short form*,

2 *Long form*,

3 *Not specified*.

* *Did not include a facial affect comparison task*.

An alternative measure of identity recognition is the two-alternative forced-choice identity discrimination paradigm, in which the participant judges whether two serially presented faces are the same or different. Again, some studies reported significant impairment in schizophrenia (Martin et al., 2005; Butler et al., 2008; Shin et al., 2008; Soria Bauser et al., 2012) while others found no impairment relative to controls (Edwards et al., 2001; Johnston et al., 2010; Soria Bauser et al., 2012). Another study requiring participants to distinguish between photographs of two learned identities, person A and person B, also reported significant impairment (Baudouin et al., 2002). Again, the reason for these inconsistent findings is unclear, but given that mean age and illness duration of the patients (Kucharska-Pietura et al, 2005), and the stimuli used in these tasks (e.g., Soria Bauser et al., 2012) are known to influence performance, it is possible these factors contributed here. While it is outside the scope of the current review it would be interesting to consider the relative severity of face perception deficits a non-clinical population of individuals high in psychosis-proneness and whether these deficits predict transition to psychosis. While impairments have been seen in some non-emotional aspects of face processing in these populations before (Poreh et al., 1994), they are not reported as frequently as deficits in face emotion perception (Waldeck and Miller, 2000; Williams et al., 2007; Germine and Hooker, 2011).

## Single feature identification

Tasks that do not assess true identity discrimination, but just one aspect of facial identity have produced more consistent results. For instance, (Bediou et al., 2005, 2007, 2012) found that patients with schizophrenia have an intact ability to discriminate the sex of faces that have been digitally morphed to increase ambiguity (see Figure 1C). Two studies (Schneider et al., 1995; Kohler et al., 2000) found that patients are significantly impaired in judging the age of a face in decades (i.e., teens, twenties, thirties, etc.), while one reported poorer accuracy, but faster reaction times than controls (Schneider et al., 1998). In contrast, one study found that patients were impaired in judging if a face is older or younger than 30 (Schneider et al., 2006), while two studies found no difference (Gur et al., 2002a,b). Taken together, these findings suggest that patients with schizophrenia are not impaired in their ability to make broad judgments about the sex or age of a face, but have greater difficulty on tasks that require more fine grained judgments. This result highlights the importance of selecting the right measure when assessing face recognition in schizophrenia. True identity perception likely reflects a judgment based on complex interactions between multiple facial features, so a task assessing just one aspect of face perception (such as sex) may not be a valid indicator of a true deficit in identity discrimination.

## Identity recollection and familiarity

Finally, tasks that assess memory of—as opposed to discrimination between—faces have largely revealed significant impairment in schizophrenia (Whittaker et al., 2001; Sachs et al., 2004; Calkins et al., 2005; Silver et al., 2009; Soria Bauser et al., 2012). In contrast, patients have been shown to have an intact ability to recognize famous faces (Evangeli and Broks, 2000; Whittaker et al., 2001; Joshua and Rossell, 2009; although one study reported significant impairment: Pomarol-Clotet et al., 2010), but are less accurate in familiarity judgment of photographs of strangers and known people (i.e., their doctor's face) (Caharel et al., 2007). However, these tasks are not necessarily an indicator of pure face processing in schizophrenia because this disorder is associated with general impairments in memory and new learning (Boyer et al., 2007). Again, care should be taken when employing memory-based face recognition tasks to ensure that general impairments in memory are taken into account.

## Impaired face processing

Face information is extracted from the environment using both face-specific and more general perceptual processes (McKone and Robbins, 2011). Some argue that face processing deficits in schizophrenia indeed represent dysfunction in face-specific perceptual processes—generally referred to as “holistic face processing.” This represents a rapid, involuntary face-specific perceptual process that integrates information across the face as a whole. It includes such information as the shapes of individual features, the relative distances between them, and the contour of the cheeks and jaw (Maurer et al., 2002; McKone and Yovel, 2009). This process is specific to invariant face information, and is therefore critical for perceiving identity (McKone and Robbins, 2011). For instance, it has been demonstrated in healthy controls that holistic processing predicts an individual's ability to remember and distinguish between faces (Richler et al., 2011). Similarly, it has been shown that individuals with congenital prosopagnosia (inability to recognize faces) perform poorly on holistic processing tasks (Palermo et al., 2011).

One common means of evaluating holistic processing is with the Face Inversion Effect—observed as a reduction in face discrimination performance for inverted faces compared to upright faces (see Figure 1D). The magnitude of this effect is thought to represent a loss of holistic information crucial to face discrimination, and is disproportionately larger for faces compared to other non-face stimuli (Yin, 1969). Studies of holistic processing in schizophrenia produced varied results, with some studies reporting normal inversion effects for faces (Schwartz et al., 2002; Chambon et al., 2006; Butler et al., 2008), while others report reduced inversion effects compared to controls (Shin et al., 2008; Kim et al., 2010; Soria Bauser et al., 2012). In particular, Shin et al. (2008) reported that patients with schizophrenia were more impaired when discriminating faces that differed in configural information, rather than featural information. An electrophysiological indicator of the face inversion effect is the N170 (Eimer, 2000), a negative potential seen using electroencephalography (EEG). The N170 is reduced in patients with schizophrenia while viewing inverted faces (Onitsuka et al., 2006; Ibáñez et al., 2012), and is associated with lower scores on measures of social functioning (Obayashi et al., 2009; Tsunoda et al., 2012). These findings suggest there may be an underlying face processing abnormality that may go undetected by commonly used behavioral measures.

In a related behavioral study, Schwartz et al. (2002) employed the composite face task, which is considered to provide a more rigorous measure of holistic processing than other inversion tasks (McKone, 2008). In this task, participants are required to make decisions about the upper halves of faces while ignoring the lower halves. These face halves are either aligned to form a complete face (producing an interference effect) or misaligned (removing the interference). When the stimuli are inverted, however, the aligned faces no longer produce strong interference effects. It was found that patients with schizophrenia showed typical patterns of interference for upright faces and not inverted faces. While this study has not been repeated, it provides support for the argument that holistic processing is largely preserved in schizophrenia and appears to contradict some of the results using the face inversion effect.

## Evidence for identity processing deficits using non-face stimuli

As outlined above, a number of studies have shown impaired performance on tasks aimed to assess face-specific processing. However, a number of similar deficits seem to be apparent on tasks using non-face stimuli. For example, Soria Bauser et al. (2012) reported reduced inversion effects for cars and bodies (see Figure 1D) that mirrored their findings using face stimuli, suggesting an impairment that encompasses more than just face-specific holistic processing. An interesting comparison is also provided by research looking at gait perception. Previous research has indicated that the identity of an individual can also be extracted from an individual's gait pattern (Cutting and Kozlowski, 1977). Through the use of point-light displays (PLD; Johansson, 1973), visually impoverished stimuli provide body form and structure solely through motion cues of coordinated dots (see Figure 1E). Similar to the ERP findings regarding face processing, the N170 component has also been found in healthy individuals during visual processing of inverted PLD and static images of bodies (Stekelenburg and de Gelder, 2004; Jokisch et al., 2005). Loula et al. (2005) demonstrated that healthy subjects exhibited superior performance in identifying self and friend's movement when compared to a stranger's movement. Furthermore, inverting the PLD resulted in chance performance across all three conditions. Unfortunately the use of dynamic gait stimuli in the investigation of true identity recognition deficits in schizophrenia has yet to be conducted. Individuals with schizophrenia are impaired in discriminating PLD-presented body movements (biological motion) from scrambled PLD body movements (Kim et al., 2005). It is therefore, conceivable that the ability to use the information provided in the point-light displays to extract identify information would also be impaired.

## Does impaired identity processing reflect a generalized attentional deficit?

One possible account for face processing deficits in schizophrenia is that they are the result of a more general impairment in allocating visuospatial attention (Baudouin et al., 2002). One suggestion is an impairment in global vs. local visual processing. “Global processing” refers to the ability to attend to *any* visual stimulus as a “whole,” as opposed to its component features (Tan et al., 2009). Studies of schizophrenia have revealed impairments in global processing, but largely preserved local processing both for static (Goodarzi et al., 2000; Silverstein et al., 2000; Johnson et al., 2005; Poirel et al., 2010) and dynamic stimuli (Chen et al., 2003). In addition, patients with schizophrenia demonstrate a bias toward attending to the local level of a stimulus, even when task demands favor a global strategy (Landgraf et al., 2011).

It is possible that a global processing deficit could contribute to impairments in identity recognition because the important global-level information is not being processed efficiently. For instance, it has been shown that identity recognition performance is improved when healthy participants are primed to adopt a global processing strategy, and impaired when primed with a local processing strategy (Macrae and Lewis, 2002; Perfect, 2003). Patients with schizophrenia similarly showed less of a reduction in identity recognition performance compared to controls when configural cues were removed from a face (Joshua and Rossell, 2009), indicating that these individuals relied more strongly on local features when identifying famous faces. Global processing deficits could also explain the expected deficits in identity recognition from gait in individuals with schizophrenia. Kim et al. (2005) argued that deficits in biological motion perception in individuals with schizophrenia may arise due to their well-documented difficulties in global motion perception (for review see Chen, 2011).

## Does impaired face identity processing reflect a general visual perceptual difficulty?

Individuals with schizophrenia show a gamut of visual perceptual impairments (see Butler et al., 2008 and the editorial of this research topic!). These difficulties include form processing such as object recognition, grouping, perceptual closure, and visual context (Place and Gilmore, 1980; Saccuzzo and Braff, 1986; Rief, 1991; Kerr and Neale, 1993; Rabinowicz et al., 1996; Kohler et al., 2000; Silverstein et al., 2000; Doniger et al., 2002; Brenner et al., 2003; Uhlhaas et al., 2006; Kurylo et al., 2007; Yang et al., 2013). Moreover, neuroanatomical data indicate that the visual cortex in schizophrenia is abnormal with respect to the density of neurons (Selemon et al., 1995), total number of neurons (Dorph-Petersen et al., 2007) and GABA concentration in the visual cortex that is associated with orientation-specific center-surround suppression (Yoon et al., 2010). Interestingly, the face fusiform area (FFA) seems relatively intact, at least functionally (Yoon et al., 2006). Given the exhaustive list of basic visual perceptual deficits in schizophrenia, it seems likely that processing of complex visual stimuli such as faces would also be compromised. Thus, it is likely that at least some aspects of face processing deficits observed in schizophrenia arise from visual cortical abnormalities.

## Conclusions

Deficits in face processing have frequently been observed in patients with schizophrenia. In order to fully understand the mechanisms underlying these impairments it is important to consider the relative contribution of the multiple factors that may be involved. The fact that deficits have been seen in face identity tasks without an emotional/expression recognition component suggests that these deficits are unlikely to be limited to emotion processing. Moreover, the observation of more generalized impairments in visual and attentional function in these patients also raises questions about whether there is indeed anything special about faces at all. Lastly, the potential role of medication in these impairments has yet to be clearly determined. It is only through future controlled studies that balance difficulty across memory, attentional and perceptual demands—or directly assess the capacities—that we will begin to understand how face processing deficits emerge in these patients.

### Conflict of interest statement

The authors declare that the research was conducted in the absence of any commercial or financial relationships that could be construed as a potential conflict of interest.
